# Algorithms for enhancing public health utility of national causes-of-death data

**DOI:** 10.1186/1478-7954-8-9

**Published:** 2010-05-10

**Authors:** Mohsen Naghavi, Susanna Makela, Kyle Foreman, Janaki O'Brien, Farshad Pourmalek, Rafael Lozano

**Affiliations:** 1 Institute for Health Metrics and Evaluation, University of Washington, Seattle, Washington, USA

## Abstract

**Background:**

Coverage and quality of cause-of-death (CoD) data varies across countries and time. Valid, reliable, and comparable assessments of trends in causes of death from even the best systems are limited by three problems: a) changes in the *International Statistical Classification of Diseases and Related Health Problems *(ICD) over time; b) the use of tabulation lists where substantial detail on causes of death is lost; and c) many deaths assigned to causes that cannot or should not be considered underlying causes of death, often called garbage codes (GCs). The Global Burden of Disease Study and the World Health Organization have developed various methods to enhance comparability of CoD data. In this study, we attempt to build on these approaches to enhance the utility of national cause-of-death data for public health analysis.

**Methods:**

Based on careful consideration of 4,434 country-years of CoD data from 145 countries from 1901 to 2008, encompassing 743 million deaths in ICD versions 1 to 10 as well as country-specific cause lists, we have developed a public health-oriented cause-of-death list. These 56 causes are organized hierarchically and encompass all deaths. Each cause has been mapped from ICD-6 to ICD-10 and, where possible, they have also been mapped to the *International List of Causes of Death *1-5. We developed a typology of different classes of GCs. In each ICD revision, GCs have been identified. Target causes to which these GCs should be redistributed have been identified based on certification practice and/or pathophysiology. Proportionate redistribution, statistical models, and expert algorithms have been developed to redistribute GCs to target codes for each age-sex group.

**Results:**

The fraction of all deaths assigned to GCs varies tremendously across countries and revisions of the ICD. In general, across all country-years of data available, GCs have declined from more than 43% in ICD-7 to 24% in ICD-10. In some regions, such as Australasia, GCs in 2005 are as low as 11%, while in some developing countries, such as Thailand, they are greater than 50%. Across different age groups, the composition of GCs varies tremendously - three classes of GCs steadily increase with age, but ambiguous codes within a particular disease chapter are also common for injuries at younger ages. The impact of redistribution is to change the number of deaths assigned to particular causes for a given age-sex group. These changes alter ranks across countries for any given year by a number of different causes, change time trends, and alter the rank order of causes within a country.

**Conclusions:**

By mapping CoD through different ICD versions and redistributing GCs, we believe the public health utility of CoD data can be substantially enhanced, leading to an increased demand for higher quality CoD data from health sector decision-makers.

## Background

Timely, valid, and reliable information on causes of death by age and sex is a critical input into public health planning, program implementation, and evaluation. Most high-income and many middle-income countries have the benefit of a complete vital registration system in which the vast majority of deaths get a certificate of death completed by a physician [1]. These information systems should in principle provide public health communities in each country with critical information needed to guide their programs. Nevertheless, analyzing levels and trends in causes of death, even in countries with well-functioning cause-of-death registration systems, remains challenging for a number of reasons related to the process of completing death certificates and the coding of each death certificate following standardized international rules.

Even with a physician-completed death certificate, assignment of the underlying cause of death can be problematic. In the Second Annual Report of the Registrar General of Great Britain in 1840, William Farr presented the statistics of causes of death (CoD), defined as "diseases, which terminate in the extinction of existence," but Farr highlighted the concern that "...the attention of the observer was less attracted to this class of facts, and overlooking the proximate cause, that is, the internal morbid process..." In that report, he also criticized the use of vague categories like "sudden death," "natural death," "visitation of God," and "old age," but he admitted that in some cases, no particular cause of death could be identified [2]. All these criticisms remain relevant today.

Analysis of cause-of-death data is intimately linked to the evolution of the *International Statistical Classification of Diseases and Related Health Problems *(ICD). Originally known as the *International List of Causes of Death*, the modern era for the ICD began when the World Health Assembly approved the sixth revision of the ICD in 1948 [3]. The new classification sought to establish an international standard for terminology and nosological criteria to attribute disease names and classify pathologies. Adoption of the ICD by the World Health Organization (WHO) also included a commitment by Member States of WHO to report national statistics based on the ICD. ICD-6 also included the adoption of an international medical certificate of CoD, an international agreement about the underlying cause of death (UCD) as the main cause to be tabulated and the rules for selecting UCD.

Despite the adoption of an international death certificate, the principle of identifying the UCD, and a standard list of causes codified in the revisions of the ICD, at least three problems create issues of comparability for public health analysis among participating countries. First, each time there is a change in the ICD, the set of causes and the codes assigned to each underlying cause change substantially. Producing time series of cause-of-death data requires mapping for some coherent set of causes across revisions - a practice often known as bridge coding [4,5]. For example, to produce a time series spanning the 20^th ^century, one would need to map across the *International List of Causes of Death *(ILCD 1-5) to the *International Statistical Classification of Diseases and Related Health Problems *(ICD 6-10). Whereas the ILCD had only been used to classify mortality, the ICD expanded to include both mortality and morbidity, thus increasing the number of causes from 179 to 20,000 [6]. Time series analyses [7-9] for selected causes have attempted to map national ICD revisions over time, but idiosyncratic national use of the ICD has limited more general approaches to bridge coding that are applicable across all countries. In addition, in the WHO database documentation [10], there is no mention of the ICD sixth revision, but during the period 1949-1957, at least 40 countries used this version and sent data to the Pan American Health Organization(PAHO) and WHO.

Second, due to the increase in the number of causes, tabulation lists were introduced starting with ICD-6. These lists provide a much shorter set of aggregate codes intended to facilitate cause-of-death reporting in countries with more limited capacity and for communication purposes. A substantial component of historical vital registration data is only available for these tabulation lists, including ICD-7 Tabulation A and B, ICD-8 Tabulation A and B, Basic Tabulation List (BTL) in ICD-9, and mortality tabulation in ICD-10. As with any aggregation procedure, substantial information is lost as compared to the fully disaggregated ICD data that were used to create these lists. For some causes, such as cardiomyopathy, pericarditis, endocarditis, and myocarditis (in BTL and ICD-7 Tab A), or source of burning and exposure to inanimate or mechanical forces in ICD-10 Tabulation list 1, assessing time trends requires some way of breaking down the tabulated data into component causes.

Third, with the advent of the sixth revision, the ICD has been used not only to code deaths by underlying cause of death but also to code other types of medical information, such as reasons for admission to or discharge from a hospital. The introduction of multiple purposes for the ICD has lead to the addition of many codes for causes that should not be considered underlying causes of death. WHO has recognized this problem by producing lists of ICD codes under the heading "List of conditions unlikely to cause death" in the appendix of Volume 2 of the second edition of the ICD [3]. Despite these recommendations from WHO, these codes are frequently used as underlying causes of death. More generally, some ICD codes are used to assign cause of death that are likely misclassifications from a public health perspective.

In 1996, Murray and Lopez [11] introduced the term "garbage coding" for the practice of assigning deaths to causes that are not useful for public health analysis of cause-of-death data as part of the assessment of the Global Burden of Disease (GBD). While some practitioners may object to the term "garbage code" as pejorative, alternative terms have not yet caught on in the literature. We follow this practice and use the term garbage code (GC) to refer to all deaths assigned to codes that should be redistributed to enhance the validity of public health analysis. The variable use of GCs across countries and over time profoundly limits meaningful comparisons of causes of death; for this reason, WHO and other analysts have sought to reassign deaths coded to GCs to other causes following various methods [11-16].

Given the importance of cause-of-death data for public health analysis, we attempted in this paper to build on prior cause-of-death analysis work [1,7,17-25] and to create a more detailed approach to these problems of comparability of ICD-coded cause-of-death data. Our goal was to maximize the public health utility of cause-of-death data. To achieve this, we created a public health cause-of-death list building on the Global Burden of Disease Study, mapped this cause list across ICD revisions, and provided a comprehensive framework for identifying and redistributing deaths assigned to GCs. We illustrated this approach using an extensive database of publicly available cause-of-death data for more than 100 countries spanning 1950 to 2008.

## Methods

### Data Sources

**Figure 1:**
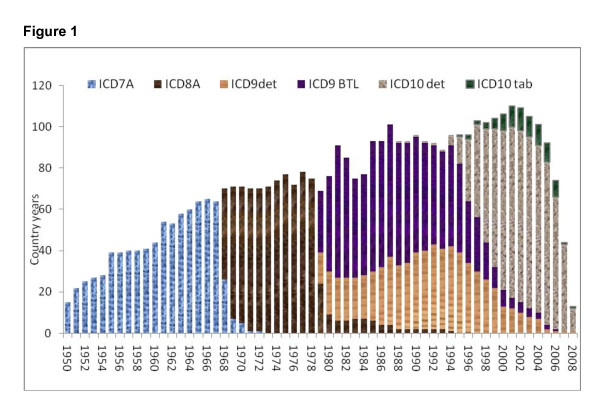
**Number of country-years of cause-of-death data by ICD revision from 1950 to 2008 used in this study based on publicly available datasets**. Between 1950-1957, 40 countries used ICD-6 and sent data to WHO using the same Tab A format as ICD-7. ICD-6 has 235 country-years and 50.9 million deaths.

In this study, we illustrated the challenges and proposed solutions to enhance comparability using a database we constructed of publicly available vital registration data coded according to various revisions of the ICD. In some cases, such as China or India, data are available only for a sample of deaths from sample registration systems or for subnational areas. In total, we had data for 4,434 country-years covering the time period 1901 to 2008 for 145 countries. These country-years of observation include 743 million deaths. Table 1 summarizes the number of country-years and deaths available for each version of the ICD in the database used for this analysis (see Additional file 1 for more detail). Figure 1 shows the number of country-years in the database by ICD revision. While many countries switch versions of the ICD soon after the release of the new version, the figure illustrates how it can take many years for all countries to change. For example, in 1994, four versions of the ICD were in use at the same time.

**Table 1:** Country year and number of deaths in study data by ICD format, 1950-2008.

ICD Format	Country years	Number of deaths (millions)
ILCD 1-5	92	26.8

ICD 6 and ICD 7 Tab A	816	146.2

ICD 8 Tab A	877	125.3

ICD 9 detail	1021	113.9

ICD 9 BTL	52	14.3

Special Country ICD 9 Tab	668	160.0

ICD 10 detail	824	123.4

ICD 10 Tab	54	21.7

Special Country ICD 10 Tab	30	12.1

**Total**	**4434**	**743.7**

Between 1950-1957, 40 countries used ICD-6 and sent data to WHO using the same Tab A format as ICD-7. ICD-6 has 235 country-years and 50.9 million deaths.

### Cause List for Analysis

The starting point for our analysis in this paper was the cause list for which we wanted to produce meaningful comparisons over time and across communities. We have taken advantage of the ongoing GBD Study. This large-scale collaboration with more than 800 scientists has developed a cause-of-death list meant to inform public health analysis. The cause-of-death list has 56 causes in three levels. Given the changes in the ICD and the complexities of GCs in different revisions, it is not possible to track all causes of death across multiple ICD revisions. Based both on the availability of detailed data and the evidence of consistency in time trends, we have been able to map 56 causes over most revisions of the ICD since 1950.

Additional file 2, Table S1 provides the cause list and ICD-10 codes for each cause. Four criteria were used to develop this list: a) causes that based on current knowledge (such as the GBD Study) are important causes of burden or are important for public health policy because they are major sources of health expenditures; b) causes that can be effectively traced across ICD-7 to ICD-10; c) causes that most often can be identified in tabulated versions of the ICD revisions; and d) the set of causes at the same level of the hierarchy that are mutually exclusive and collectively exhaustive. The cause list is organized hierarchically such that at the most aggregate levels, there are three broad groups of causes, and under each level of aggregation, there are more detailed causes. We organized the substructure of the list to allow for maximum comparability over time and assigned unique codes to facilitate analysis by others using our software.

For each of the 56 causes shown in the list, we mapped across the various revisions of the ICD, including back to ICDL-1 through ICDL-5 and the various national versions of ICD revisions and tabulation lists. Additional file 3 shows whether or not a cause can be traced through the various revisions of the ICD for each cause on our list. Examination of the list shows, for example, that all the CoD can be traced through ICD-7 to ICD-10 in the detailed lists, but some causes cannot be traced in the ICD-7 and 8 Tabulation list B or in ICD-9 country-specific lists used in China and India.

### A Typology of Garbage Codes

In addition to identifying a cause list and mapping this cause list across various revisions of the ICD, the largest impediment to comparability is the presence of a different set of GCs in each ICD revision. To more fully understand the problem of garbage codes, we created a typology of these codes that distinguishes four types of GCs. This typology has been developed taking into consideration the following: the likelihood that a condition can be an underlying cause of death; the need for codes that provide a location for unspecified or ambiguous causes of death; and the need for codes that represent causes that are not underlying but intermediate or final events in the chain leading to death. Four categories were identified:

1. *Causes that cannot or should not be considered as underlying causes of death*. These are codes that are included in the ICD because of its use for classifying health service encounters but that do not signify underlying cause of death. Examples of this type of GC are all the codes under chapter 18 of ICD-10 or R codes. This category also includes two special cases in the cardiovascular area: essential primary hypertension and atherosclerosis. Essential primary hypertension is included in the ICD to classify clinical encounters, but for most physicians, it should be considered a risk factor for cardiovascular disease and not the underlying cause. This distinction between what is a risk factor and what is an underlying cause is somewhat arbitrary but necessary to enhance comparability across revisions. Finally, we included in this category a number of causes that are described as the long-term sequelae of disease, such as G82, paraplegia and tetraplegia, or O94, sequelae of complication of pregnancy, childbirth, and the puerperium. In these cases, for public health purposes, it is more useful to assign these deaths to the underlying cause despite the long time lag between disease and death.

2. *Intermediate causes of death such as heart failure, septicemia, peritonitis, osteomyelitis, or pulmonary embolism*. These are clearly defined clinical entities, but each has an underlying cause that would have precipitated the chain of events leading to death. Physicians who have not been adequately trained in the principles of the ICD underlying cause of death often use these causes on death certificates.

3. *Immediate causes of death that are the final steps in a disease pathway leading to death*. Examples of this include disseminated intravascular coagulation or defibrination syndrome (D65). The pathway to death includes the final immediate cause, an intermediate cause, and the underlying cause that triggered the chain of events. Cardiac arrest (I46) and respiratory failure, not elsewhere classified (J96), are other examples.

4. *Unspecified causes within a larger cause grouping*. For many diseases, such as neoplasms, a code is included within the grouping for an unspecified site. This is an illustration of a GC that is not important for assessing aggregate deaths from neoplasms from all sites but is important when assessing site-specific death rates. Another important example is the injury category in which some injuries are coded to unspecified factors or intent.

Table 2 provides a listing of the number of each type of GC that we identified related to our 56-cause list. The largest category of GCs is type 1. Assessment of the number of GCs, especially in category 4, is a function of the level of detail in the final cause list that is being developed.

**Table 2:** List of garbage codes for ICD-10 based on the public health analysis cause list of 56 causes.

GC Type	ICD-10 Codes
Type 1	A31.1, A59, A60.0, A71-A74, A63.0, B00.0, B07, B08.1, B08.8, B30, B35-B36, F32-F33.9, F40-F42.9, F45-F48.9, F51-F53.9, F60-F98.9, G43-G45.9, G47-G52.9, G54-G54.9, G56-G58.9, H00-H04.9, H05.2-H69.9, H71-H80.9, H83-H93, J30, J33, J34.2, J35, K00-K11.9, K14, L04-L08.9, L20-L25.9, L28-L87.9, L90-L92, L94, L98.0-L98.3, L98.5-L98.9, M03, M07, M09-M12, M14-M25, M35.3, M40, M43.6-M43.9, M45.9, M47-M60, M63-M71, M73-M79, M95-M99, N39.3, N40, N46, N60, N84-N93, N97, Q10-Q18, Q36, Q38.1, Q54, Q65-Q74, Q82-Q84, R00-R99, B94.8, B949.9, G80-G83, Y86, Y87.2, Y89, I10, I15, I70

Type 2	A40-A41, A48.0, A48.3, E85.3-E85.9, E86-E87, G91.1, G91.3-G91.8, G92, G93.1-G93.6, I26, I27.1, I44-I45, I49-I50, I74, I81, J69, J80-J81, J86, J90, J93, J93.8-J93.9, J94, J98.1-J98.3, K65-K66, K71-K72 (except K71.7), K75, K76.0-K76.4, K92.0-K92.2, M86, N14, N17-N19

Type 3	D65, I45-I46, J96

Type 4	C80, C26, C39, C57.9, C64.9, C76, D00-D13, D16-D18, D20-D24, D28-D48, A49.9, B83.9, B99, E88.9 I51, I99, X59, Y10-Y34

### Redistributing Deaths Assigned to GCs

To enhance comparability, we followed the conceptual approach developed by Murray and Lopez in the GBD and currently applied by WHO; namely, to reassign deaths from GCs to causes in our cause list. This approach can be divided into three steps: identify GCs, identify the target causes where the deaths assigned to a GC should in principle be reassigned (based on pathophysiology or an assessment of certification practice); and choose the fraction of deaths assigned to a GC that should be reallocated to each target cause. In the work to date, the identification of target causes for a GC has been based on very general groupings, such as all injuries or all Group I diseases, and the allocation algorithm has largely been based on proportionate distribution within an age-sex group.

We expanded the approach taken in the literature. First, we carefully considered pathophysiology in identifying target causes for a GC. For example, for peritonitis, our targets include digestive diseases, such as intestinal obstruction; genitourinary diseases such as salpingitis and oophoritis; pregnancy, childbirth, and puerperium disease; conditions such as abortions; and some intentional and unintentional injuries. Details for some examples (exposure to unspecified factor X59, female genital organ malignant neoplasm, unspecified site C57.9, heart failure I50, peritonitis K65, septicemia A40, A41) are provided in Additional file 4 to give further illustration of this approach.

Second, we distinguished three methods for assigning GC deaths to a set of target underlying causes: proportionate redistribution within an age-sex group, statistical models, and expert judgment. We used a combination of all of these approaches depending on the four types of GCs. For causes with little information content, we used proportionate redistribution across target causes. In the case of heart failure, we developed a statistical model that helps identify the proportion of deaths for each target code within a given age-sex group. The algorithm eliminates all deaths with the code HF (ICD-10 I50) from the database. It identifies the fraction that should be extracted from HF and assigned to each of the target categories. To estimate the fractions allocated to each target code, we regressed by age, sex, and development status using all available ICD-10 mortality data the fraction of heart failure deaths from all deaths related to heart failure, including target causes.

Finally, for many GCs, we reviewed the published literature and engaged in consultation with GBD expert groups to develop an expert-based algorithm for assigning the fraction of deaths assigned to a GC within an age-sex group to be allocated to different target causes. A further criterion used in developing these expert algorithms was to compare the time trends in a cause by country across various revisions of the ICD. For example, the distribution of GCs to target codes for heart failure is a function of local epidemiology. Redistribution of GCs should in principle generate more plausible or continuous time trends commensurate with the underlying nature of a cause without observing the major discontinuities associated with a change in ICD.

The algorithms for reassigning each of the GCs have been developed in Stata. While conceptually simple, the allocation of each GC to target causes for each age-sex group is computationally intensive. We intend to make our software available to researchers or government agencies to enhance the comparability of their own data. We are currently producing a usable version of the program code for the general public. Once complete, the software will be publicly available on the Web site of the Institute for Health Metrics and Evaluation.

## Results

### Evolution of garbage codes across ICD revisions

**Figure 2:**
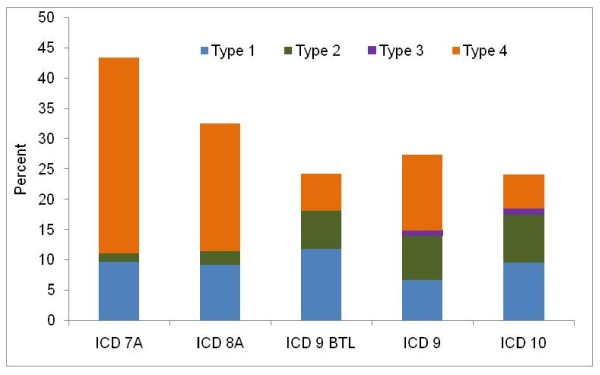
**Percentage of garbage codes by type of GCs and ICD version, all ages**. Between 1950-1957, 40 countries used ICD-6 and sent data to WHO using the same Tab A format as ICD-7. ICD-6 has 235 country-years and 50.9 million deaths.

Figure 2 illustrates the fraction of all deaths in our cause-of-death database that have been assigned to the four types of GCs in various revisions of the ICD. The fraction of deaths assigned to GCs type 1 through 3 has, in general, been stable over the past 50 years, with between 10% and 18% assigned to these types. In the ICD-7 and 8 Tabulation lists, a large fraction of deaths is classified as belonging to GC type 4. This includes deaths grouped into aggregate codes, such as all malignant neoplasms that had to be reassigned to site-specific locations. As such, these aggregate codes exaggerate problems with ICD-7 Tabulation A and ICD-8 Tabulation A, which are driven by the use of summary tabulations rather than a preponderance of certification using unspecific or ambiguous codes. The slight increase in the fraction of all deaths assigned to GCs in ICD-10 reflects more countries reporting cause-of-death data, including many developing countries, and the increased complexity and number of codes in the latest revision.

**Figure 3:**
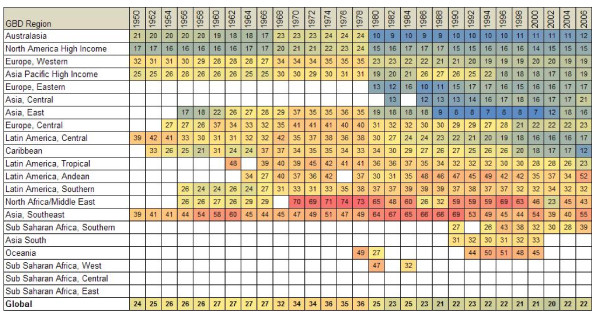
**Fraction of all deaths coded to GCs for all years available in each GBD region by year**.

Figure 3 provides information on the fraction of GCs by GBD region over time (see Additional file 5 for the list of countries by GBD region, including a link to how regions are defined). Australasia consistently has the lowest fraction, but in all the high-income countries, the fraction of deaths assigned to GCs has in general been declining. Given the more extensive definition of GCs in this analysis as compared to prior work on the GBD, the percentages appear higher. North Africa, the Middle East, and Southeast Asia have the highest levels of GCs, often exceeding 50% of all deaths in a given year.

**Figure 4:**
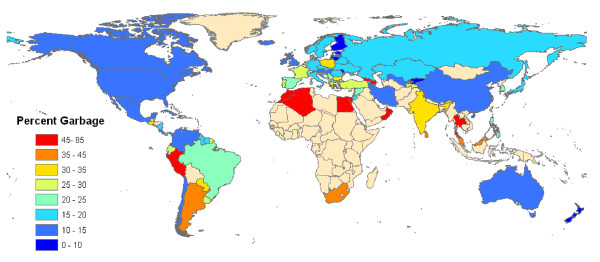
**Fraction of deaths assigned to GCs in the latest ICD-10 year since 2000**.

The percentage and pattern of GCs differ over time in each country. These differences are related to the knowledge and education of medical doctors or coroners performing the diagnosis of cause in mortality, the education of coders, the different algorithms in the ACME (if this type of software is used), the ICD format (detail or tabulation in ICD-9 or ICD-10), and the format of published or shared data. The countries with the highest fraction of GCs are Thailand and Egypt. Figure 4 shows the fraction of deaths assigned to GCs in the latest year of ICD-10 data available. Many developing countries, such as Oman, Egypt, Peru, Georgia, and many countries in North Africa and some in South Asia, have very high levels of GCs. Countries with a stable and well-established death registry system usually have low or medium levels of GCs. There are a few developing countries with stable death registry systems and low percentages of GCs, such as Chile, Mexico, and Cuba.

**Figure 5:**
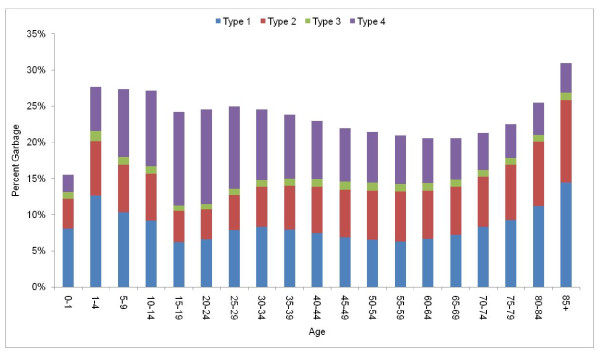
**Percentage of all deaths coded to GCs by age in all country-years of ICD-10 data**.

The fraction of deaths assigned to GCs varies substantially by age (Figure 5). Type 1 GCs increase gradually with age - larger numbers of deaths at the oldest ages where diagnostic detail may be absent may account for this general trend. Type 2 GCs also increase and even more markedly with age, perhaps reflecting the increasing complexity of identifying underlying causes across age in some cases, especially due to heart failure. Finally, type 4 GCs have a different age pattern. This category includes cases where there is some ambiguity about the exact underlying cause but the death clearly belongs to a particular group of causes.. In particular, the larger fraction of deaths falling under this category at young ages can be traced to a substantial number of injury deaths for which full detail is not available. As injuries account for a larger fraction of deaths at younger ages, this explains the larger share of Type 4 at these age groups.

### Impact of Redistribution of GCs on mortality profile

**Figure 6:**
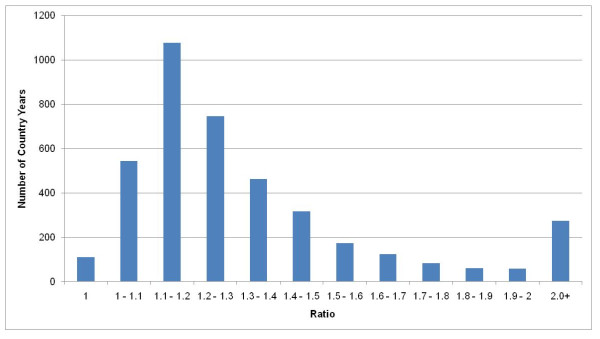
**Ratio of the number of deaths from maternal causes after redistribution to the number of deaths before GC redistribution across 4,434 country-years of ICD-coded mortality data**.

The impact of redistributing GCs can be seen in three ways: the change in the number of deaths assigned to a cause; changes in the cause composition across ages; and changes in the time trend in specific causes. Figure 6 illustrates, for example, the ratio of the number of deaths due to maternal causes before and after the redistribution of GCs. Across country-years in the database, these ratios range from 1 to 5, with an average value of 1.4, implying that GC redistribution increases vital registration maternal deaths by 40% on average. These findings are consistent with the published literature on maternal mortality audits that find this ratio to be around 0.9 - 2.0, which provides some external validation of this approach [26-35].

**Figure 7:**
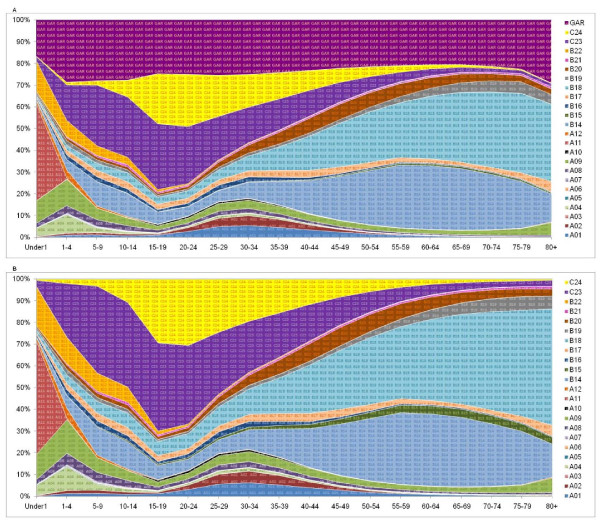
**A & 7B Distribution of all ICD-10 coded cause-of-death data for 878 country-years by age and 24 causes before and after GC redistribution**. A01 - Tuberculosis in all type, A02 - HIV/AIDS, A03 - Sexually transmitted diseases except HIV, A04 - Intestinal Infectious, A05 - Vaccine Preventable, A06 - Malaria, A07 - Parasitic and Vector born disease, A08 - Meningitis/Encephalitis/Hepatitis, A09 - Respiratory Infections, A10 - Maternal conditions, A11 - Neonatal conditions, A12 - Nutritional deficiencies, B14 - Neoplasms, B15 - Diabetes, B16 - Endocrine, nutritional, blood, and immune disorders, B17 - Mental/behavioral and neurological conditions, B18 - Cardiovascular and circulatory diseases, B19 - Respiratory diseases, B20 - Digestive diseases, B21 - Genitourinary/skin/musculoskeletal diseases, B22 - Congenital anomaly, C23 - Unintentional injuries, C24 - Intentional injuries, GAR - Garbage

Figure 7a shows the distribution across 24 causes (we collapsed 56 causes for illustration purposes) in our hierarchical cause tree before redistribution by age for all 162 million deaths (874 country-years) in ICD-10 format in our database (we collapsed the 56 cause-list to 24 causes for illustration purposes). Figure 7b shows the pattern of mortality for the same set of 162 million deaths after application of the redistribution methods for all the GCs in ICD-10. Because many GCs have specific targets and redistribute by age and sex separately, the fraction of deaths assigned to a cause changes differentially by cause, age, and sex. For example, there is little change in the fraction of deaths assigned to malignant neoplasms but large changes in the number of deaths assigned to cardiovascular diseases and injuries in some ages. Also, because these redistributions have been done at the country level, if we make the graph by region or countries, these patterns will be different and will be related to the GC variations by age and sex in that country or region.

**Figure 8:**
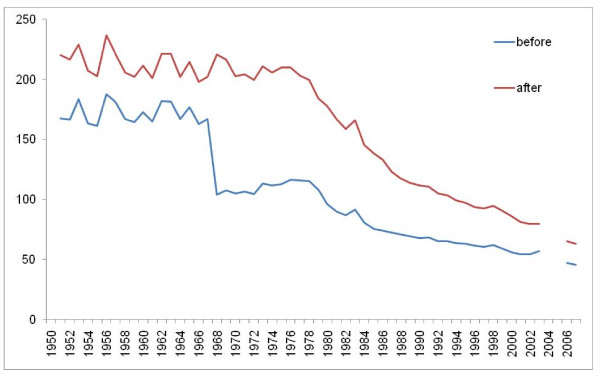
**Age-standardized death rate for ischemic heart diseases in Italy before and after GC redistribution**.

**Figure 9:**
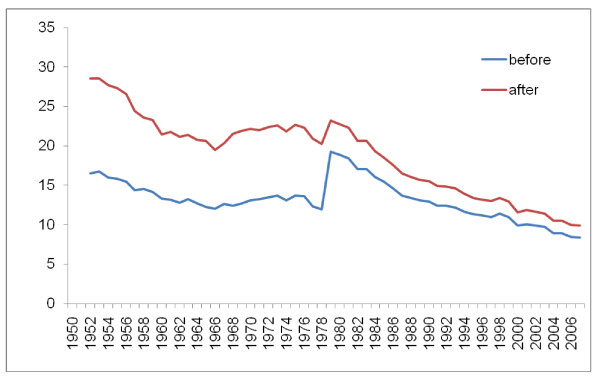
**Age-standardized death rate for all digestive disease except cirrhosis in France before and after GC redistribution**.

**Figure 10:**
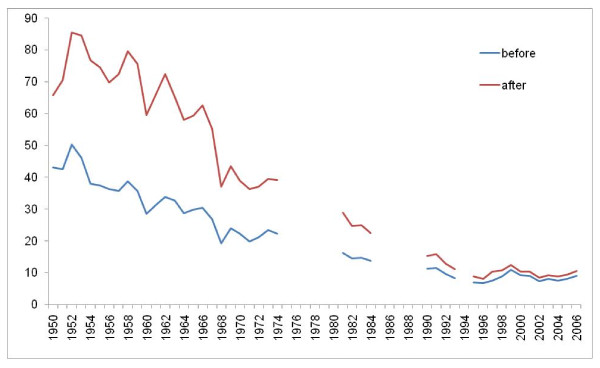
**Age-standardized death rate for nutritional deficiencies in El Salvador before and after GC redistribution**.

**Figure 11:**
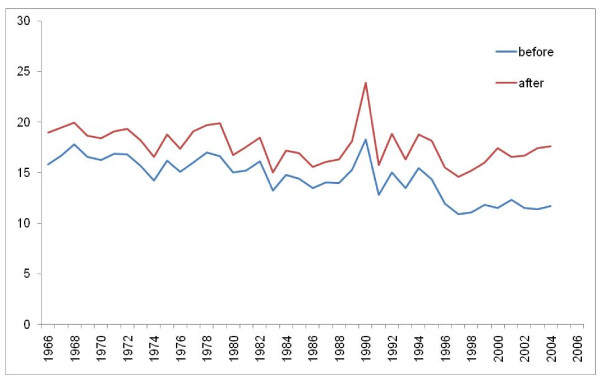
**Age-standardized death rate for transport injuries in Bulgaria before and after GC redistribution**.

Figures 8, 9, 10, and 11 illustrate the impact of GC redistribution process on time trends for particular causes in specific countries. The blue lines represent a cause mapped across different versions of the ICD before the reallocation of garbage codes. Figure 8 highlights how major shifts in reported numbers of deaths from ischemic heart disease can be addressed through redistribution of GCs. The time trend for Italy after redistribution appears to be plausible and consistent with the general decline in IHD seen in many places starting in 1970. Figure 9 shows for all digestive diseases excluding cirrhosis how a major shift that occurred with the introduction of ICD-9 in France does not appear once GCs have been adequately addressed. Figure 10 uses an incomplete time series for a middle-income country, El Salvador, to show the impact of GC redistribution. The decline in the age-standardized death rate from nutritional deficiencies is much more noticeable after redistribution. Finally, Figure 11 illustrates how redistribution of some injury codes suggests that transport injuries in Bulgaria are actually increasing rather than slowly declining, particularly in the last 10 years.

## Discussion

In this study, we have extended work undertaken as part of the GBD Study and by WHO to provide tools to enhance the public health use of cause-of-death data. For a list of 56 causes, we mapped across ICD-7 Detail through ICD-10. We have identified four types of GCs in all versions of the ICD and country-specific cause-of-death lists. For each of these GCs, we have identified likely codes for which these deaths should ideally be assigned based on pathophysiology or certification practice. Practical algorithms to redistribute these deaths have been developed and implemented using statistical software. These algorithms have been applied to a database of more than 700 million ICD-coded deaths that are available from public sources covering more than 4,000 country-years. Based on our results, we believe that these algorithms can be generally applied to country-level ICD data by analysts interested in comparability over time and place. Through the application of these approaches, we believe that the public health utility of cause-of-death data can be substantially enhanced, leading to increased demand for higher quality cause-of-death data from health sector decision-makers.

These CoD analysis algorithms affect our interpretation of trends and the relative rankings of countries for selected causes. For example, if we compare country-by-country rankings of the age-standardized death rate for ischemic heart disease (83 countries in 2005), the effect of GC redistribution is to change the rank of 19 countries by two to four ranks and 49 countries by five or more ranks. Similar findings hold true across nearly all causes. For example, for deaths due to transport injuries, 21 countries change by two to four ranks and 51 countries by five or more ranks). Perhaps even more importantly, for some major noncommunicable causes, the overall effect of mapping and GC redistribution is to change the trend over time. As noted as long ago as 1976 [36], the timing of the epidemiological transition is substantially influenced by the correction of GCs and bridge coding.

In this work, we have looked in much greater depth at the likely target causes to which GCs should be redistributed and have explored three different methods for choosing the fraction in an age-sex group that should be allocated to each target GC. There is, nevertheless, a substantial scope for further research on choosing these redistribution proportions for each GC onto target underlying causes. Ideally, for validation, one would like to collect a dataset where the "true" underlying cause is known based on autopsy or extensive clinical records but the deaths have been assigned to a GC in the normal course of death registration [37-39]. This, however, is unlikely to occur because most deaths with an autopsy or extensive clinical records are not assigned to GCs on their death certificate. Ex post studies are hard to conduct because the records required to ascertain underlying cause may not have been collected or be available [40]. Nevertheless, innovative methods such as matching or blinded death certification may be applicable to the challenge of putting the GC redistribution algorithms on a stronger empirical footing. An important area for research will also be to try and characterize the uncertainty in the redistribution algorithms so that this uncertainty can be reflected in the adjusted death rates for a cause in a particular country and year.

Figure 4 shows that the fraction of deaths assigned to GCs across countries is highly variable even in the latest year of data availability. If all countries had the resources and policy commitment to achieve the levels of quality seen in New Zealand or Australia [20], the quality of cause-of-death data around the world would be dramatically improved. While WHO undertakes important efforts to help countries implement ICD revisions, the global health community has invested little in helping countries more effectively implement cause-of-death certification and coding. For public health analysis, we believe that it would be useful to clearly communicate to physicians who are going to complete death certificates that certain causes of death should not be used because they either cannot be underlying causes of death or are immediate or intermediate causes of death. Application of the algorithms in this study may help national authorities to demonstrate the extent of garbage coding and therefore motivate further action at the local level to improve the quality of certification [41,42].

While we have made substantial efforts to consistently map a limited set of important causes of death across the various revisions of the ICD and to deal with the challenge of GCs in each revision, many problems remain. Inconsistencies among the ICD eighth revision and other revisions were not totally solved. The capacity to reconstruct reasonable sequences for ICD-7- and ICD-8-coded data is more limited due to the fact that much of the data are reported using limited tabulation lists.

We distinguish mapping across revisions of the ICD to maximize comparability from formal dual coding of a set of deaths according to two different revisions of the ICD. Such formal bridge coding studies are available for a few select countries and a limited number of ICD revision changes. Comparable cause-of-death statistics, however, require the more general approach of mapping across revisions of the ICD. We recognize the problems associated with applying a universal algorithm across all countries but have designed our choice of causes in the cause list and mapping over the revisions of the ICD to facilitate comparisons wherever possible.

Beyond its incursion into other areas of health care that go beyond the statistics of mortality, the ICD remains the global standard reference frame for describing and analyzing major health problems in society. Efforts such as this to enhance the utility of this information for public health analysis should highlight the intrinsic value of vital registration data with standardized death certification and ICD coding. The ICD and the work of WHO to revise and maintain the classification is a true international public good that requires ongoing support from the global health community.

## List of Abbreviations

BTL: Basic Tabulation List (ICD-9); CoD: Cause of Death; GBD: Global Burden of Disease (Study); GC: Garbage Code; ICD: International Statistical Classification of Diseases and Related Health Problems; IHME: Institute for Health Metrics and Evaluation; ILCD: International List of Causes of Death; UCD: Underlying Cause of Death; WHO: World Health Organization; ACME: Automatic Classification of Medical Entry.

## Competing interests

The authors declare that they have no competing interests.

## Authors' contributions

MN led the project, including statistical analysis, interpretation of results, and writing the first draft the paper. SM, KF, and JO undertook construction of the software for mapping ICD codes, garbage redistribution packages, and implementation. FP interpreted data and helped write the first draft. RL guided the analysis and interpretation of data and wrote the second draft. All authors have read and approved the final manuscript.

## Supplementary Material

Additional file 1 **Annex 1**. Details of causes-of-death data by country and source. Click here for file

Additional file 2 **Table S1**. Cause of death list for public health analysis with associated ICD-10 Click here for file

Additional file 3 **Annex 2 **Tracing each cause in the 56-cause list through the various revisions of the ICD. Click here for file

Additional file 4 **Annex 3**. Details of redistribution packages for exposure to unspecified factor X59, female genital organ malignant neoplasm, unspecified site C57.9, heart failure I50, peritonitis K65, and septicemia A40, A41. Click here for file

Additional file 5 **Annex 4**. GBD regions list and link to how regions are defined. Click here for file
