# Supplementary material for: Algorithms for enhancing public health utility of national causes-of-death data
Source: Popul Health Metr. 2010 May 10;8:9. doi: 10.1186/1478-7954-8-9 (PMC2873308; doi:10.1186/1478-7954-8-9)
Supplement: Additional file 2 — Table S1. Cause of death list for public health analysis with associated ICD-10 [file 1478-7954-8-9-S2.DOC]

**Table S2 -** **Cause of death list for public health analysis with associated ICD-10 codes.**
